# Encapsulation of Screen-Printed Electrolyte-Based Organic Electronic Components for Long-Term Operation in Varying Environmental Conditions

**DOI:** 10.1021/acsami.5c09639

**Published:** 2025-08-05

**Authors:** Xin Wang, Kathrin Freitag, Jessica Åhlin, Peter Andersson Ersman

**Affiliations:** Printed, Bio- and Organic Electronics − Smart Hardware − Digital Systems, 388792 RISE Research Institutes of Sweden, Södra Grytsgatan 4, Norrköping SE-602 33, Sweden

**Keywords:** PEDOT:PSS, printed electronics, encapsulation, barrier material, organic electrochromic display (OECD), organic electrochemical transistor (OECT), printed adhesive

## Abstract

With the advancement of printing techniques, material choices, and ink development, high-performance printed electrochemical components relying on organic conducting polymers are beginning to mature and find traction in numerous applications in different areas, such as organic electrochromic displays (OECDs) and logic circuits based on organic electrochemical transistors (OECTs). However, the inherent hygroscopic nature of various materials in the devices, combined with electrochemically dictated working mechanisms, makes the devices sensitive to environmental changes, such as relative humidity (RH) levels and temperature (*T*). To ensure reliable operation of the devices, there is a need to mitigate the influence of ambience. In this article, we use commercially available printable adhesives and plastic substrates with predeposited barrier coatings to provide device encapsulation. The developed process allows for tight and conformal sealing along the topography of printed conductors reaching out from the encapsulated devices, thereby blocking the most probable leakage path. Consequently, the device performance with respect to environmental fluctuations is maintained, and the best barrier materials ensure that the performances of screen-printed OECDs and OECTs remain intact after storage in harsh conditions, such as the combination of low RH and low *T* or high RH and high *T*. Satisfactory results are achieved after a storage time of 1 week in each condition; 10% RH and 10 °C, 80% RH and 20 °C, and 90% RH and 40 °C. Additional tests performed for even longer storage times and harsh conditions, 90% RH and 40 °C, and extremely dry environment (<3% RH and 20 °C), respectively, showed devices with excellent switching performances upon evaluation. It was also discovered that the color retention of OECDs switched to their colored state, after 1 week of storage in open-circuit mode at different environmental conditions, was considerably improved upon encapsulation with barrier films.

## Graphical abstract

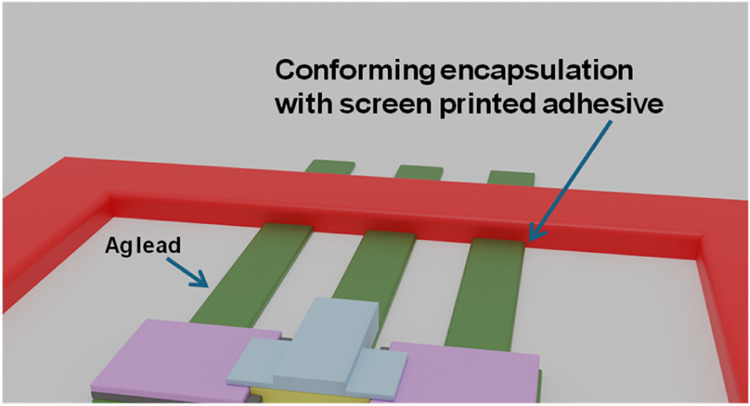

## 1 Introduction

Considerable progress has been achieved for organic, printed, and flexible electronics based on conducting polymers in recent years. A few prominent examples of printed organic electronic components that are important for electronic systems are organic light emitting diodes (OLED),^1^ organic photovoltaics (OPV),^2,3^ organic field effect transistors (OFET),^4^ organic electrochromic displays (OECD),^5–9^ and organic electrochemical transistors (OECT).^10,11^

The strength and advantage of printed organic electronics originate from their inherent flexibility and possibility of solution processing at low temperatures. Thus, printable components and systems with adequate performance can be achieved on flexible polymer- and paper-based substrates. However, one drawback with organic electronic materials is that they usually are sensitive to variations in the environmental conditions, e.g., oxygen and moisture, upon storage as well as during operation.^12–14^

To overcome this challenge, various encapsulation materials and sealing techniques have been investigated. Glass is the ultimate encapsulation material due to its excellent barrier properties combined with maintained transparency, and it may even be manufactured into thin flexible sheets or rolls.^15,16^ Several successful encapsulation attempts have been reported by laminating printed electronic components between glass panes.^17,18^ However, depending on whether thin or thick glass is used, this results in either brittleness or rigidity and heavy weight. Relatively thick organic polymer films, e.g., polyvinyl butyral (PVB) and ethylene vinyl acetate (EVA),^19^ have also been used as encapsulants, for example laminated on solar cells, but their barrier performance is usually limited.

Instead of bulky glass or thick polymer sheet materials, barrier materials can be applied as coatings. Broadly, there are two types of encapsulation strategies when using barrier materials, i.e., lamination of a barrier film prefabricated on a carrier substrate or *in situ* deposition of the barrier material on top of the device by, e.g., vacuum-assisted deposition. Unless the encapsulation layers are deposited *in situ*, an adhesive material is needed to seal the edges of the encapsulated devices.

Various inorganic and organic materials have been extensively investigated as barrier coatings or thin films on different substrates and structures, either as single layers or as a stack of multiple layers. The multiple layers can consist of different inorganic coatings, or different organic coatings, but may also be based on hybrid multilayer coatings of both inorganic and organic materials, or nanocomposites.^12,13,20–27^

Inorganic materials that have been used as the barrier layer are mainly oxides and nitrides.^13,25^ Various organic materials that have been tested include polyvinyl acetate (PVA),^28^ alucone,^29–33^ pentaerythritol triacrylate,^34^ and organosilicone.^35^ Composites of different materials have also been created and used as barriers.^28,36,37^

Different process methods for depositing the thin barrier layers include, e.g., spin coating,^34^ inkjet printing,^38^ physical vapor deposition (PVD),^33,39^ chemical vapor deposition (CVD),^40^ initiated CVD (iCVD),^35,41,42^ plasma enhanced chemical vapor deposition (PECVD),^23^ atomic layer deposition (ALD),^13,42^ and plasma enhanced atomic layer deposition (PEALD).^35,38^

Deposition of thin barrier films, or multilayered thin barrier films, directly on top of the devices has been developed extensively in recent years; however, such methods are more suitable for very thin devices without major changes in topography.^22^ Lamination using polymer-based barrier films or precoated barrier layers is a more viable and practical method for additive manufacturing, e.g., screen-printing, on large-area substrates. For this reason, several flexible barrier film materials are commercially available.^43–47^

Different encapsulation/lamination architectures using precoated barrier films applied on plastic substrates have also been evaluated, where the adhesive can be applied either globally or just around the edge. For flexible devices using polymer substrates, when using flexible barrier films, the encapsulation can be applied on both sides or only on the print side if one of the barrier films is used as the substrate for the deposition of the printed materials. Encapsulation by laminating a barrier film with a precoated global (full coverage) adhesive layer, e.g., pressure-sensitive adhesive (PSA), hot melt, or a global flexo-coated epoxy layer, has been demonstrated even in a roll-to-roll large-area encapsulation process for OPV devices.^48^ In addition, encapsulation by attaching a barrier film with a dispensed adhesive layer circumventing the device (edge seal) has also been demonstrated.^13,22,49^

Edge-sealing adhesive materials that have been tested include epoxy,^23,49^ polyisobutene (PIB),^50–54^ UV-curable adhesives, UV epoxy,^55,56^ glass frits,^57^ SURLYN ionomers,^55^ etc. Thick edge-sealing materials with high water vapor transmission rate (WVTR) values are not desirable since they allow for a large amount of water vapor penetration due to the combination of high WVTR and increased cross-sectional area. Epoxy-based materials are typically not screen-printable and therefore difficult to upscale. In addition, epoxy-based materials may also cause severe chemical reactions with conjugated polymers. As far as we know, no printable edge-sealing process to allow for scalable manufacturing has yet been demonstrated.

OLED and OPV devices require dry preconditioning and a higher level of encapsulation, i.e., barrier materials with very low WVTR values. For OECD and OECT devices, since they contain an electrolyte material, it is important to keep the ionic mobility at a certain level. This implies a requirement for proper initial conditioning of the components at an optimized relative humidity (RH) level prior to encapsulation. Hence, successfully encapsulated OECDs and OECTs will both keep the initial moisture content within the devices and prevent additional moisture ingress into the devices.

The conducting polymer poly­(3,4-ethylenedioxythiophene):poly­(styrenesulfonate) (PEDOT:PSS) is used as the active material in OECDs and OECTs reported herein.^58^ The OECDs can be used to visually understand the environmental impact on the switching performance in organic electrochemical devices. The working principle for the PEDOT:PSS-based OECD is that it switches to dark blue color upon reduction, while oxidation switches PEDOT:PSS to an almost transparent state; the latter appears white in the reflective OECDs reported herein due to a white and opaque electrolyte. The OECD is sensitive to environmental storage conditions. Due to this, nonencapsulated or inadequately encapsulated samples may exhibit three different appearances when they are reduced, determined by the sharpness and color intensity of the reflective display segments. One is the desired state exhibiting deep coloration and sharp edges of the display segments; for nonencapsulated samples, this state is usually obtained at room temperature and intermediate RH levels; see Figure 1a. The second state is defined by prolonged switching time and low color contrast due to an incomplete electrochromic switch; for nonencapsulated samples, this state typically occurs at low temperatures and dry environments (Figure 1b). The third state is defined by blurry edges of display segments and a short color retention time in open-circuit mode, which occurs when the OECDs are exposed to elevated temperatures and high RH levels (Figure 1c). Changes in the environmental conditions imply different moisture levels within nonencapsulated devices, and this is the explanation to the three different appearances upon electrochromic switching; it is especially the PEDOT:PSS and the electrolyte layers that are sensitive to environmental fluctuations.

**Figure 1:**
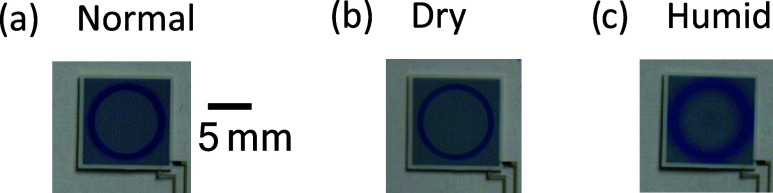
(a) Desired state (normal; 45% RH and 20 °C), exhibiting high color contrast and sharp OECD segment edges, obtained at room temperature and intermediate RH levels. (b) Low temperature and low RH levels result in prolonged switching time and lower color contrast due to incomplete electrochromic switching (dry; 10% RH and 10 °C). (c) High temperature and high RH levels result in blurry segment edges and short retention time of the reduced OECD segments (humid; 90% RH and 40 °C). The scale bar in panel (a) is valid also in panels (b) and (c).

To enable addressing of OECDs and OECTs by external electronic circuitry, printed conductors, e.g., silver or carbon, are typically printed at a thickness of 5–10 μm and a line width of, e.g., 200 μm with a 400 μm pitch between the conductors. This poses a challenge for the encapsulation using a global double-sided precoated adhesive approach since it is difficult to achieve a perfect and conforming edge sealing where these conductors exit the device to reach the external electronic circuitry. This nonconforming configuration forms an “air pocket” along the conductors. This is because the encapsulation layers (the barrier film and the global sealing material) are too thick for the line width, thickness, and pitch of the printed conductors reaching out from the device. The double-sided adhesives are available in various thicknesses, typically ranging from 20 to 150 μm, and the barrier film (thin inorganic barrier material coated on a plastic substrate) will have an additional thickness that typically spans in the range of 50–200 μm.^43–45^

To exemplify the air pocket problem, the switching behavior of encapsulated OECDs using global double-sided adhesives on the barrier film upon storage in very humid environment is shown in Figure 2. In normal conditions, the OECD exhibits sharp contrast when switched from its neutral state (Figure 2a) to its dark blue colored reduced state (Figure 2b). However, upon exposure to very humid conditions for a few days, a switching behavior with blurry segment edges can be observed in these encapsulated OECDs relying on a global sealing material; Figure 2c shows that the problem is initiated in the segments located closest to the printed conductors at the bottom of the OECD, followed by moisture propagation further into the encapsulated device (Figure 2d). Figure 2e illustrates the air pocket phenomenon that has been identified as the cause of moisture ingress. This problem also brings a design dependence when comparing different devices since the pitch of the printed conductors will have a large impact on the air pockets created upon lamination of the barrier film (including the global double-sided adhesive) on top of the printed conductors.

**Figure 2:**
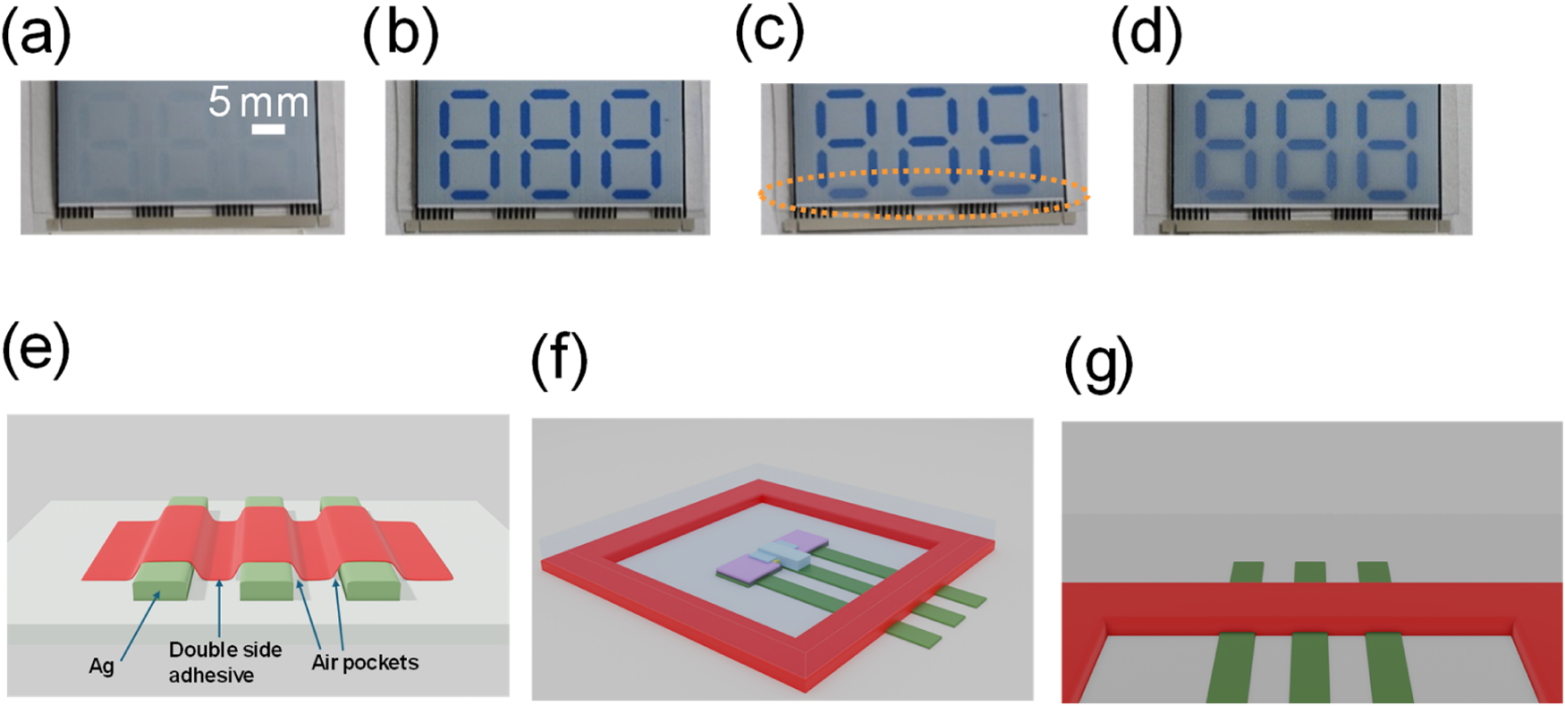
Photographs of OECDs encapsulated with barrier films with precoated double-sided adhesive layers are shown in panels (a–d), showing the problem with a nonconforming global adhesive layer. (a) Appearance of the noncolored (OFF) state of the encapsulated OECD is shown. (b) Switching the OECD to its blue colored (ON) state at room temperature and ∼45% RH results in an appearance with high color contrast and sharp segment edges. (c) OECD has been stored in a hot and humid environment (90% RH and 40 °C), which results in moisture ingress through the air pockets that are created along the printed conductors. This is evidenced by the weak color contrast and blurry appearance in the OECD segments located closest to the printed conductors (marked with orange color in the image). The upper segments are still sharp since the moisture has not yet reached this part of the OECD. (d) Longer storage time in hot and humid environment results in moisture propagation further into the device, as evidenced by the blurry edge appearance also in several other OECD segments. (e) Air pockets are formed alongside the printed conductors when using a barrier film with a global double-sided adhesive tape. (f) Screen-printed adhesive frame circumvents the device structure, followed by lamination of the barrier film. (g) Zoom-in image of the screen-printed adhesive layer, which mitigates the air pocket problem. Note that the barrier film is omitted in panels (e) and (g). The scale bar in panel (a) is valid also in panels (b–d).

To overcome the air pocket problem occurring when using the global double-sided adhesive layer in the encapsulation process, a conformable adhesive layer is needed to ensure proper edge sealing between the barrier film serving as the substrate, the printed conductors, and the laminated barrier film. A printed adhesive layer should be a viable option,^59^ but the approach of using printable edge-sealing materials for the encapsulation has, until now, barely been explored. In this work, we present an encapsulation process that is compatible with the screen-printing process used for the fabrication of OECDs and OECTs.^6^ The screen-printed OECD and OECT components are manufactured either directly on the barrier film or on a poly­(ethylene terephthalate) (PET) substrate. A screen-printable adhesive material is used to join the devices with the barrier film in the next lamination step. The printed adhesive effectively fills the spaces between the printed conductors, thereby preventing the formation of air pockets along the printed conductors that are required to establish the connection between the devices and the external address electronic circuits. The schematic in Figure 2f,g shows the use of a screen-printed adhesive layer as the edge-sealing material, such that the air pockets are effectively averted due to the conformity of the printed adhesive material.

## 2 Experimental Section

Screen-printing of the devices has been carried out on both ordinary PET substrates and different barrier films, and the encapsulation is completed by lamination of different barrier films using screen-printed adhesive frames. The devices are always encapsulated with the same kind of barrier material on both sides. The encapsulation process is simplified when the devices are printed directly on the barrier material since only the print side needs an extra process step to finalize the encapsulation. When instead using PET as the substrate for the printed devices, barrier film lamination is required on both sides of the PET to obtain properly encapsulated devices.

### 2.1 Materials

Most of the materials used for the screen-printing process of OECD and OECT devices have been described previously.^6^ Briefly, PEDOT:PSS (SV4 from Heraeus), carbon (7102 from DuPont), insulator (5018 from DuPont), silver (CXT-0644 from Sun Chemical), and electrolyte (E003 from RISE) are the screen-printing inks that are required for the manufacturing process. Additionally, KIWOPRINT UV 92, a UV-curable adhesive purchased from KIWO, was used as the screen-printable adhesive material for the lamination step that completes the encapsulation process.

PET (Polifoil purchased from Policrom) is an ordinary substrate material, which also is used as a reference encapsulation material, despite its poor barrier properties. Three different PET films with barrier coatings have been used: Opteria MS-F0050PAC (henceforth referred to as LINTEC-3) and Opteria MS-F2050PAC (henceforth referred to as LINTEC-4) from LINTEC Corporation, and Ultrabarrier 510-F from 3M. The PET and barrier material properties are listed in Table 1. In addition to the larger thickness, the 3M barrier film possesses a more complex multilayer structure, with a fluoropolymer on one side and PET on the other side and the barrier material embedded as a middle layer. For the LINTEC barrier films, the structure is simpler since the barrier layer surface is exposed without any extra protection layer.

**Table 1:** Specifications of the Barrier Materials

Barrier material	Polifoil (PET used as reference; short name: PET)	Opteria MS-F0050P (barrier/PET; short name: LINTEC-3)	Opteria MS-F2050P (barrier/PET; short name: LINTEC-4)	3M 510-F (PET/barrier/PET/fluoropolymer; short name: 3M)
Supplier	Policrom	LINTEC/Opteria	LINTEC/Opteria	3M
WVTR (g·m^–2^·day^–1^)	4	6.0 × 10^–3^ @ 40 °C, 90% RH (measured by aquatran-2 E398-03)	5.0 × 10^–4^ @ 40 °C, 90% RH (measured by aquatran-2 E398-03)	<6 × 10^–5^ @ 50 °C, 100% RH (measured by mocon aquatran analyzer)
Total substrate thickness (μm)	125	50	50	200
OTR (cm^3^·m^–2^·day^–1^)	1.3 ^60^	<0.05	<0.05	∼0.001 (50 °C, 100% RH)
Optical transmittance (%)	90	91	88	>90
Haze (%)	1.3 ^61^	1	1	2.8 ^62^

### 2.2 Design of Device Architectures

In order to focus on the evaluation of the encapsulation process, the OECD and OECT device designs used herein are similar to previously reported screen-printed devices.^6^ Figure 3a–c shows the OECD and OECT device architectures.

**Figure 3:**
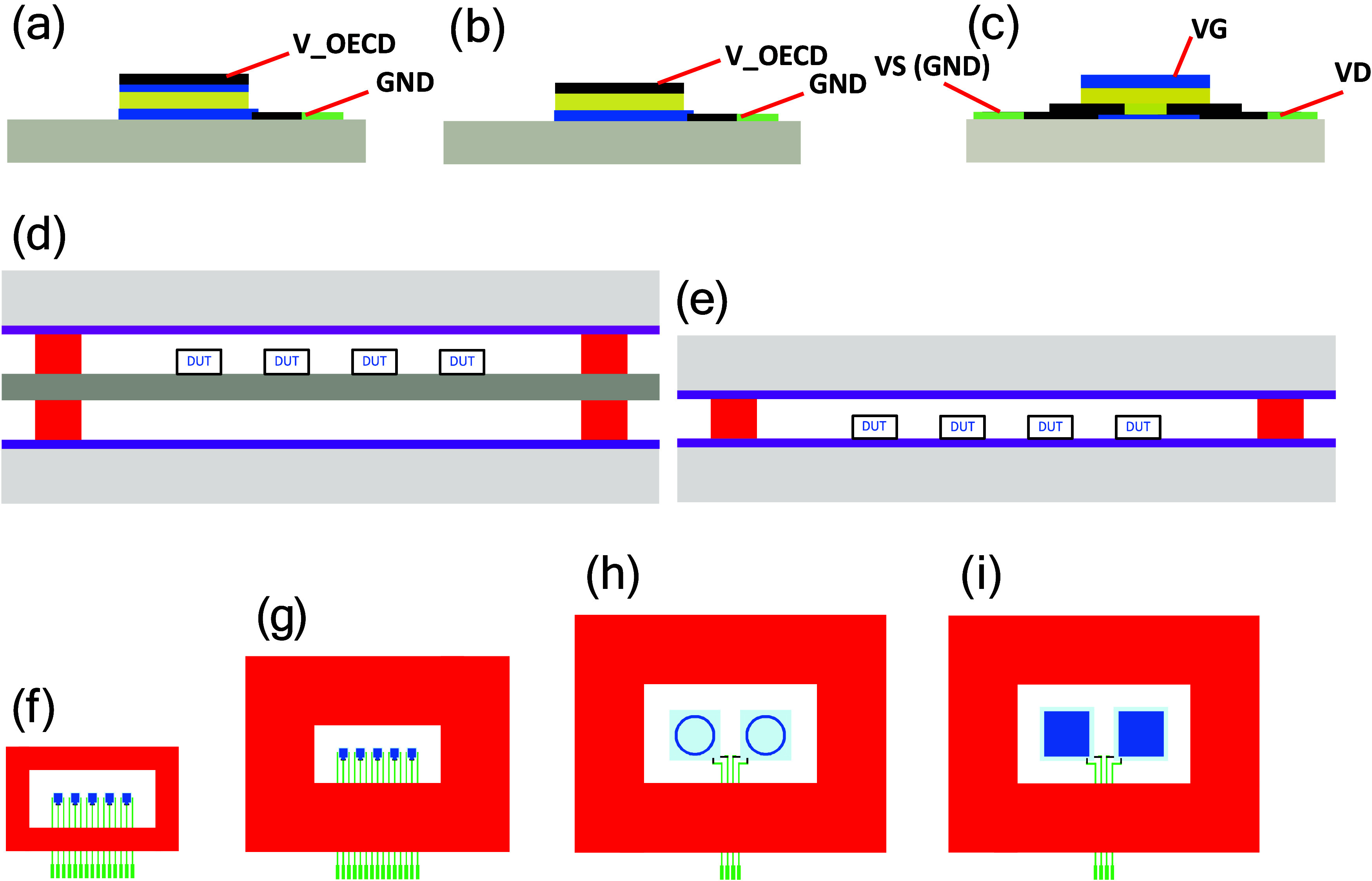
(a) Cross-sectional view of the OECD with PEDOT:PSS as the counter electrode. (b) Cross-sectional view of the OECD with carbon as the counter electrode. (c) Cross-sectional view of the OECT architecture. (d) Cross-sectional view of encapsulated devices, where the devices under test (DUT) are printed on a PET substrate and encapsulated with barrier films laminated on both sides. (e) Cross-sectional view of devices screen-printed directly on the barrier film; hence, the encapsulation is completed by laminating the barrier material only on the side with the printed structures. (f) Top view of five OECTs with a 5-mm-wide screen-printed adhesive layer. (g) Top view of five OECTs with a 15-mm-wide screen-printed adhesive layer. (h) Top view of two OECDs with ring-shaped segments with a 15-mm-wide screen-printed adhesive layer. (i) Top view of two OECDs with square-shaped segments with a 15-mm-wide screen-printed adhesive layer. The pitch of the silver lines is 1250 μm in all of the screen-printed OECD and OECT device structures, and 200 μm line width and 1050 μm separation is used at the location of the screen-printed adhesive layer. The screen-printed adhesive layer is used for the lamination of the barrier film to complete the device encapsulation process. Color codes: blue—PEDOT:PSS; yellow—electrolyte; black—carbon; green—silver; gray—substrate; red—adhesive; purple—barrier; turquoise—insulator defining active areas, i.e., the switchable OECD segments are marked in dark blue color.

For the OECT, the switchable PEDOT:PSS channel area is 200 × 200 μm^2^. For the evaluation of transistor devices, five OECTs with identical design were grouped together and encapsulated in one enclosed unit. For the OECD, two different OECD segment patterns were used: one is ring-shaped and the other is square-shaped. The ring-shaped segments are used to evaluate the switching behavior after storage under different environmental conditions. The square-shaped segments are used for tracking the retention behavior of the color contrast after storage in open-circuit mode under different environmental conditions. The diameter of the ring-shaped segment is 9 mm, and its switchable area is approximately 20 mm^2^, while the square-shaped OECD segment has a switchable area of 100 mm^2^. Two OECD devices, with either ring- or square-shaped segments, are included in one encapsulated unit, where one of the OECDs has PEDOT:PSS as both the bottom (electrochromic) and top (counter) electrodes, while PEDOT:PSS and carbon serve as the electrochromic electrode and the counter electrode, respectively, in the other OECD.

To achieve proper device encapsulation, the screen-printed adhesive is deposited as a frame that encircles the device structure, thereby also covering silver-based conductors that are screen-printed underneath. In this work, the width of the screen-printed adhesive layer is either 5 or 15 mm.

Two different configurations have been used to achieve proper device encapsulation when barrier coatings are used on transparent plastic films. The device is printed on an ordinary PET substrate in one of the configurations, and the device is encapsulated from both sides afterward, as shown in Figure 3d. In the other configuration, the device is printed directly onto the barrier-coated PET substrate, and the encapsulation is then completed by only laminating a barrier film on the side with the printed structures; see Figure 3e. Top view schematic of the OECD and OECT structures, including also the screen-printed adhesive layer (5 or 15 mm wide), are shown in Figure 3f–i.

### 2.3 Screen-Printing of the Adhesive Layer and the Encapsulation Process

First the OECDs and the OECTs were manufactured according to the previously described screen-printing process,^6^ either on the PET substrate or directly on the barrier film.

To encapsulate the devices, a 5- or 15-mm-wide double layer (screen-printed twice with a curing step after each printing step) of the KIWOPRINT UV 92 adhesive material was screen-printed as a frame encircling each group of devices; see Figure 3f–i. A polyester-based 77-48 screen-printing was used for the deposition of the adhesive material, and each layer was UV-cured at an exposure dose of 700 mJ cm^–2^. When the barrier film also was used as the device substrate (Figure 3e), the adhesive material was screen-printed on the substrate barrier film to allow for the lamination process. When PET instead was used as the device substrate (Figure 3d), the adhesive material had to be screen-printed on the device side of the PET substrate as well as on one of the barrier films to prepare for the lamination of one barrier film on each side of the PET-based device substrate. Reference samples based on devices printed on PET substrates, followed by lamination of a PET-based “barrier film”, were also created.

After completing the screen-printing process of the devices on top of either the barrier film substrate or the PET-based substrate and the screen-printing and UV-curing of the adhesive layers, the final lamination step is performed to complete the encapsulation process.

The lamination of the barrier films was performed on a Lamiart 470LSI. The barrier films provided by LINTEC (50 μm thick) were laminated at 70 °C with a throughput speed of 33 cm min^–1^, while the 3M barrier films (200 μm thick) were laminated at 90 °C at a speed of 17 cm min^–1^.

Encapsulation of screen-printed OECTs directly on the thin (50 μm) LINTEC barrier films turned out to be difficult, probably due to the generation of defects during encapsulation. The printed features of the OECT devices are relatively small, and this, in combination with the device topography and the thinness of the barrier film, could cause damage on both the OECT devices and the barrier coating during the lamination process. Direct printing of the OECTs on the thick (200 μm) 3M barrier substrate was however quite successful. Due to this, investigations were carried out on five different types of OECTs: reference samples with printed devices on PET and encapsulated by PET; printed devices on PET and encapsulated by LINTEC-3, LINTEC-4, and 3M barrier films on both sides, respectively; and printed devices on the 3M barrier film and encapsulated by 3M. For OECDs, however, there was no problem to encapsulate devices screen-printed directly on all barrier films, including also LINTEC-3 and LINTEC-4.

### 2.4 Device Characterization and Environmental Tests

Prior to the long-time storage tests in various environmental conditions, reference measurements of the encapsulated OECDs and OECTs were recorded in an ambient environment before the samples were put into the climate chamber.

For the weeklong storage tests of the OECTs and OECDs, the environmental conditions and sequences were 45% RH and 20 °C (ambient environment); 10% RH and 10 °C; 80% RH and 20 °C; 90% RH and 40 °C. The samples were stored in the designated environment for 1 week before they were brought into the ambient environment for temperature equilibration, after which the measurements were carried out.

For the OECTs, the transfer characteristics of the transistors were measured. The transfer characteristics of the OECTs were measured by keeping the voltage applied at the drain electrode (*V*_D_) constant at −1 V, while sweeping the gate voltage (*V*_G_) from 0 to 1.5 V and then back to 0 V. *V*_G_ was changed in steps of 10 mV at a sweep rate of approximately 50 mV s^–1^.

For OECDs, current vs time characteristics during color switching of the OECDs were used to follow up any changes of their switching behavior after storage in different environmental conditions. For switch time measurements, the OECD devices were first oxidized to decolor them completely, by applying −2 V to the counter electrode for 4–6 s, followed by recording the current as a function of time when reducing the OECDs to the blue state by applying 3 V for 10 s. Ring-shaped OECD segments with 15-mm-wide adhesive frames, screen-printed directly onto the barrier films, were used for these storage tests.

The square-shaped OECDs were used for the color retention tests, i.e., the ability of the OECD to maintain its colored state when kept in the open-circuit mode. OECD samples with 15 mm printed adhesive frames were used for the study, and carbon was used as the counter electrode in these OECD architectures. To measure the color contrast and investigate the color retention behavior, the color coordinates (CIELAB) and the color contrast (ΔE*) were determined using spectrophotometer (Mercury from Datacolor).^63^ The color coordinates of the fully colored states were first measured and used as the initial reference values. To color the OECDs in this test, 3 V was applied to the counter electrode for 6 s, and the coloration of the OECDs was always carried out in an ambient environment. The fully colored samples were then put into the climate chamber for conditioning at 10% RH and 20 °C for 1 week, followed by immediate measurements of the color coordinates after bringing the samples out from the chamber. The procedure of switching the OECDs to their fully reduced blue colored states, climate chamber storage for 1 week followed by measurements of the color coordinates of the residual color, was performed also for the two remaining environmental conditions in this test; 80% RH and 20 °C as well as 90% RH and 20 °C.

## 3 Results and Discussion

Seamless encapsulation, free from air pockets, was achieved by the combination of screen-printed adhesive materials and barrier films. The thickness of the screen-printed adhesive layer was around 26 μm, while the silver (Ag) layer thickness was around 7 μm, which resulted in conformal coverage of the Ag layer profile after the deposition of the screen-printed and UV-cured adhesive material, and this was proven to be a good strategy to obtain void free joints at the interfaces of the adhesive layers. To exemplify, photographs of the encapsulated OECTs are shown in Figure S1.

### 3.1 OECT Climate Chamber Tests

The measurement results of the environmental influence on the switching characteristics of the screen-printed and encapsulated OECTs are shown in Figure 4, after 1 week of storage at different environmental conditions. The OECTs encapsulated by using PET as the barrier film, the reference samples, were dried out when stored at low RH and low temperature (10% RH and 10 °C); hence, their current modulation capability was completely disabled. After storage at 80% RH and 20 °C, the OECTs regained their current modulation capability; however, the leakage currents from the gate electrodes increased substantially as compared to the switching behavior prior to the chamber test; see Figure S2a–j. Storage in 90% RH and 40 °C environmental conditions further deteriorated the switching performance. By encapsulating both sides of the PET substrate, which includes the printed OECTs, with either LINTEC-3 or LINTEC-4 barrier films resulted in improved OECT switching performance, as evidenced by the low impact on the device performance upon storage at 10% RH and 10 °C. These two types of samples also showed satisfying results after storage at 80% RH and 20 °C. After storage at 90% RH and 40 °C, the devices relying on the LINTEC-3 barrier film showed increased gate current levels and a threshold voltage shift. The devices encapsulated with the LINTEC-4 barrier film and the 5-mm-wide adhesive layer showed similar changes of gate currents and threshold voltages; however, the devices with the LINTEC-4 barrier film and the 15 mm adhesive layer width showed good switching behavior also in the 90% RH and 40 °C environmental condition.

**Figure 4:**
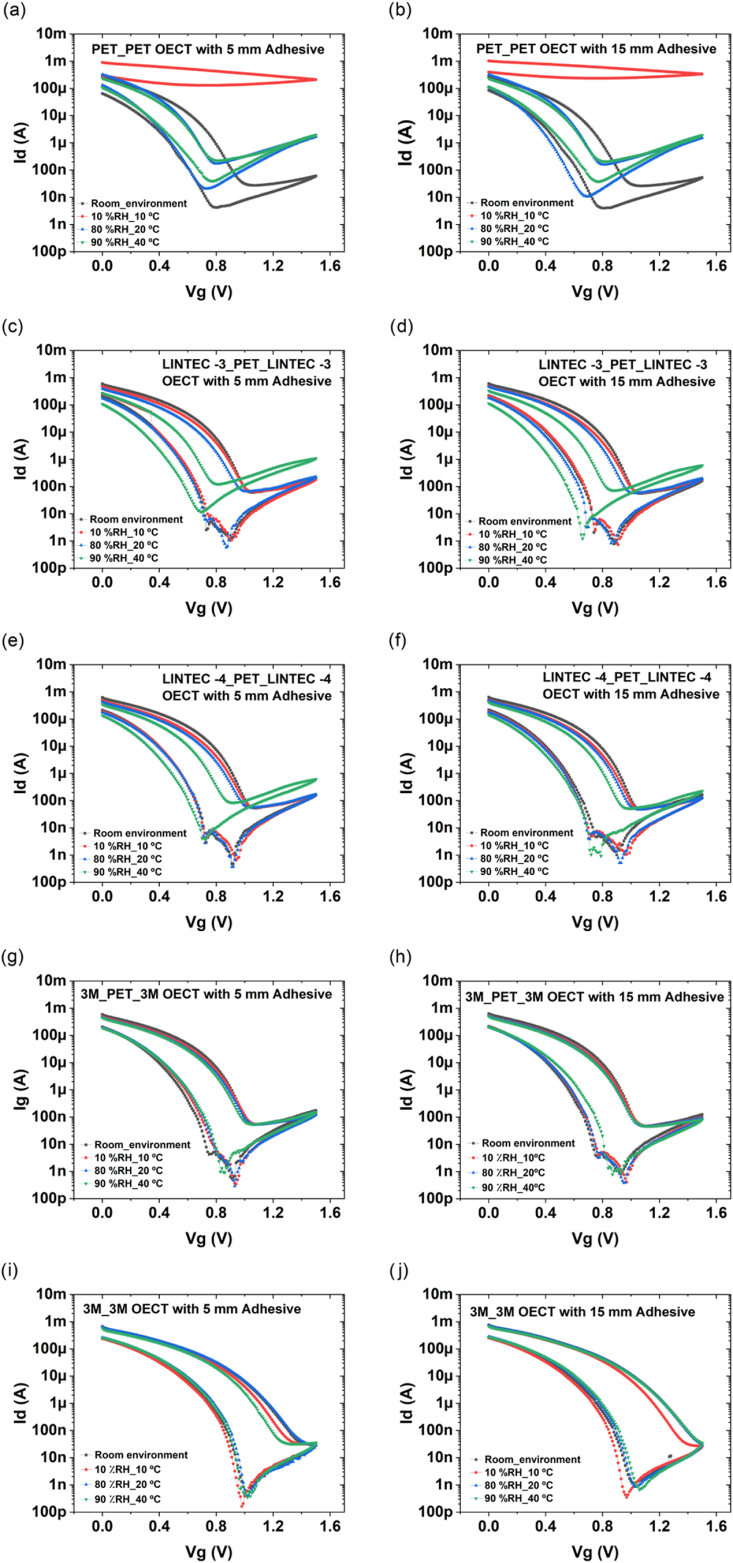
For printed devices on PET, the encapsulation has been achieved either by printing the adhesive layers followed by lamination of an additional PET film on top of the screen-printed structures (a, b) or by printing the adhesive layers followed by lamination of (c, d) LINTEC-3, (e, f) LINTEC-4, and (g, h) 3M barrier films on both sides of the PET substrate. (i, j) OECT devices have also been screen-printed directly on top of the 3M barrier film, whereafter the encapsulation was completed by printing the adhesive layers followed by lamination of another 3M barrier film on top of the printed devices. Every curve is based on the averaged data from five transistors located inside the same encapsulation unit. (a, b) For OECTs encapsulated with PET, the capability to modulate the current is completely disabled after storage in a dry environment (10% RH and 10 °C). (c, d) OECTs encapsulated with LINTEC-3 are unaffected in most of the environmental conditions; however, storage in 90% RH and 40 °C resulted in a threshold voltage shift and elevated gate current levels (Figure S2). (e, f) OECTs encapsulated with LINTEC-4 show even more similar switching behavior after exposure to different environmental conditions. Yet, these devices are to some extent affected after storage in 90% RH and 40 °C, especially the devices having a 5-mm-wide adhesive layer. (g, h) OECTs printed on PET and encapsulated with 3M show an almost identical switching behavior for all of the different environmental conditions. (i, j) OECTs printed directly on 3M and then encapsulated with another 3M barrier film also show an identical switching behavior for the different storage conditions. These devices also exhibited the lowest gate current levels.

Encapsulating both sides of the OECTs with the 3M barrier film, while still using PET as the substrate for the printed devices, resulted in even better performance of the device characteristics. The devices were performing well after storage at 10% RH and 10 °C as well as 80% RH and 20 °C, and less variation of the gate currents was also observed after storage at 90% RH and 40 °C.

Good results were also achieved for the OECTs printed directly on the 3M barrier film, followed by encapsulation of the print side with another 3M barrier film. The device performance throughout the environmental test cycle sequence showed only minor deviations when compared with the initial switching characteristics. The difference in threshold voltages observed when comparing the transfer measurement results of the two different sets of OECTs encapsulated with the 3M barrier film is explained by fluctuations of the environmental condition the samples were exposed to prior to the lamination process, since the encapsulation process of these samples was not completed at the same time.

The results show that encapsulation using the screen-printed adhesive frame as the edge sealing when laminating the barrier film is a promising route toward manufacturing of OECTs with maintained switching performance upon storage in different environmental conditions. Overall, only minor differences could be observed when comparing the 5 and 15 mm widths of the screen-printed adhesive layers, indicating that an adhesive width of 5 mm is sufficient. The main difference is that the devices with a 15 mm adhesive width showed slightly lower gate leakage current levels. In addition, the results of the samples with a 5 mm adhesive width screen-printed on different substrates/barrier films show that higher-grade barrier films give less environmental dependence, by observing their differences in switching performances upon storage in various environmental conditions. This confirms that even the 5 mm adhesive width prevents moisture ingress/egress into/from the devices and that the impact of moisture ingress/egress at the adhesive cross section is negligible. The slightly better performance in devices with a 15 mm adhesive width is explained by the longer diffusion path. The moisture ingress/egress along the edges of the encapsulated devices is determined by both the length of the diffusion path of the adhesive layer and the cross-sectional area that is exposed to the external environment. The long diffusion path of the adhesive layer, in this case equal to either 5 or 15 mm, results in a relatively low WVTR, despite the poor intrinsic barrier properties of the adhesive material. The total thickness of the printed adhesive double layer is 26 μm, resulting in a small cross-sectional area of the adhesive layer that is at least a factor of 270 times smaller than that of the barrier layer covering one side of the devices. Such a small cross-sectional area of the adhesive layer suppresses the total amount of water vapor that can diffuse through the adhesive layer to a level that is comparable with the contribution from the barrier layers covering the devices due to the relatively large area of the latter. In Figures S3 and S4 as well as Tables S1 and S2, the dimensions of the OECT device structures along with a simplified water vapor permeation analysis are given. Briefly, it is plausible that for the LINTEC-3 barrier film, the inner part of the barrier film governs the OECT switching performance; i.e., the water transmission through the adhesive is less than that through the LINTEC-3 barrier film in the middle of the device. For LINTEC-4, however, the water transmission through the barrier film appears to be similar, or even lower, than that through the adhesive, which is evidenced by the slightly better switching performance in the OECTs with a 15 mm adhesive frame, as compared to the corresponding device with 5 mm adhesive width. For the 3M barrier film, there is no significant difference when comparing the 5 and 15 mm adhesive widths, which, among the different combinations of barrier films and adhesive widths, both demonstrate the least amount of water transmission. This, in turn, could be an indication that the adhesive allows more water transmission, or possibly similar, as compared to the 3M barrier film.

It is evident that encapsulation using PET foil on top of PET substrates is insufficient, which is explained by the high WVTR value of the PET material. Encapsulation using both LINTEC-3 and LINTEC-4 resulted in dramatically improved OECT switching performance, and since the performance was slightly better for samples with LINTEC-4, it also shows that the WVTR of the barrier material is critical. OECTs encapsulated by using the 3M barrier film showed even better switching performance due to the lowest WVTR value among the evaluated barrier films, thereby confirming the importance of having low WVTR values upon storage in extreme environmental conditions.

The drain current (*I*_D_) level reaches its lowest value at an approximate *V*_G_ of 1 V in most of the curves, which indicates that the OECT channel has been fully reduced. Beyond this point, the OECT enters a regime where the parasitic gate current (*I*_G_) contribution at elevated gate voltages dictates the current recorded at the drain electrode, which explains the slowly increasing *I*_D_ observed for *V*_G_ exceeding approximately 1 V. The corresponding *I*_G_ plots for different samples tested in different environmental conditions are included in Figure S2.

The parasitic gate leakage current at a higher gate voltage is attributed to water splitting reactions, since the carbon-based source and drain electrodes connected to the PEDOT:PSS-based OECT channel are exposed to the electrolyte.^10,64,65^ By reducing the areas of the gate electrode and the electrolyte, and even more importantly also the areas of the carbon-based source and drain electrodes exposed to the electrolyte, the gate current levels can be substantially reduced; see Figure S5 for measurement results of the OECTs with decreased gate current levels.

The threshold voltage (*V*_TH_) is dependent on the environmental condition. This is due to the fact that the amount of moisture inside the device will influence the kinetics of the electrochemical reactions upon device operation. Investigation of the *V*_TH_ of a nonencapsulated OECT stored inside a climate chamber clearly shows that *V*_TH_ decreases when increasing the RH level; see Figure S6 for further details.

### 3.2 OECD Climate Chamber Tests

The encapsulated OECDs were tested to see whether the influence of various environmental conditions can be mitigated by proper encapsulation. The OECD devices were screen-printed directly on top of the respective barrier film, followed by encapsulation using the same barrier film (Figure 3e). The results show that all three types of barrier films (LINTEC-3, LINTEC-4, and 3M) worked well to prevent moisture ingress/egress when combined with the screen-printed adhesive layers. The OECDs switch without any change in sharpness and color contrast, as compared to the “normal” state before starting the environmental tests, despite the exposure to various environmental conditions for long periods of time. Hence, the barrier films in combination with the printed adhesive layers could fend off both the dry and humid environments surrounding the devices and thereby maintain the initial OECD switching performance. And as expected, the reference OECD sample “encapsulated” with a PET film was not performing well; its switching characteristics and appearance followed the external environmental conditions and exhibited the “dry” and “humid” states defined in Figure 1.

Since properly encapsulated OECDs maintain their visual appearance, in terms of sharpness and color contrast, the switching characteristics (current vs time) are used as a more sensitive probe to monitor changes in the device performance upon exposure to various environmental conditions. Figure 5 shows the switching characteristics of the ring-shaped OECD segments for different barrier materials upon long-term storage in different environmental conditions. The OECD encapsulated with PET, serving as a reference, shows that PET provides almost no protection after 1 week of storage due to its high WVTR value. For the OECDs using PET as the encapsulant, the switching behavior follows the same pattern already shown for the OECTs in Figure 4, i.e., the color contrast is weak and the switch current is low after storage at low temperature and low RH, whereas storage at high RH (at both 20 and 40 °C) results in increased switch currents but worsened color contrast due to blurry OECD segment edges. The evaluation of OECDs printed on, and encapsulated with, LINTEC-3 and 3M barrier films, respectively, resulted in that both the optical and electrical switching behavior obtained in ambient environment could be maintained also in the other environmental conditions, which clearly indicates that successful encapsulation has been achieved for these OECDs. Due to this, OECDs including the LINTEC-4 barrier film, which has even lower WVTR value as compared to the LINTEC-3 barrier film, were omitted in this test. The larger area of the printed electrolyte in OECDs, as compared to that of the OECTs (22 and 111× larger as compared to the OECTs shown in Figures 4 and S5, respectively), could more efficiently dampen the effect of humidity changes inside the encapsulated devices. These dimensional differences could explain the similar switching behavior obtained for OECDs encapsulated with LINTEC-3 and 3M, despite the difference in WVTR values when comparing these two barrier films. Note that the water permeation for encapsulated OECDs with different widths (5 or 15 mm) of the screen-printed adhesive layer is elaborated in Figures S7 and S8 as well as in Table S3; hence, the dimensional effects of the encapsulated area and the adhesive material are also considered. The data confirms that, by assuming that the electrolyte will be most affected by water transmission through the encapsulation, the water transmission per electrolyte area is much lower for OECDs than for OECTs.

**Figure 5:**
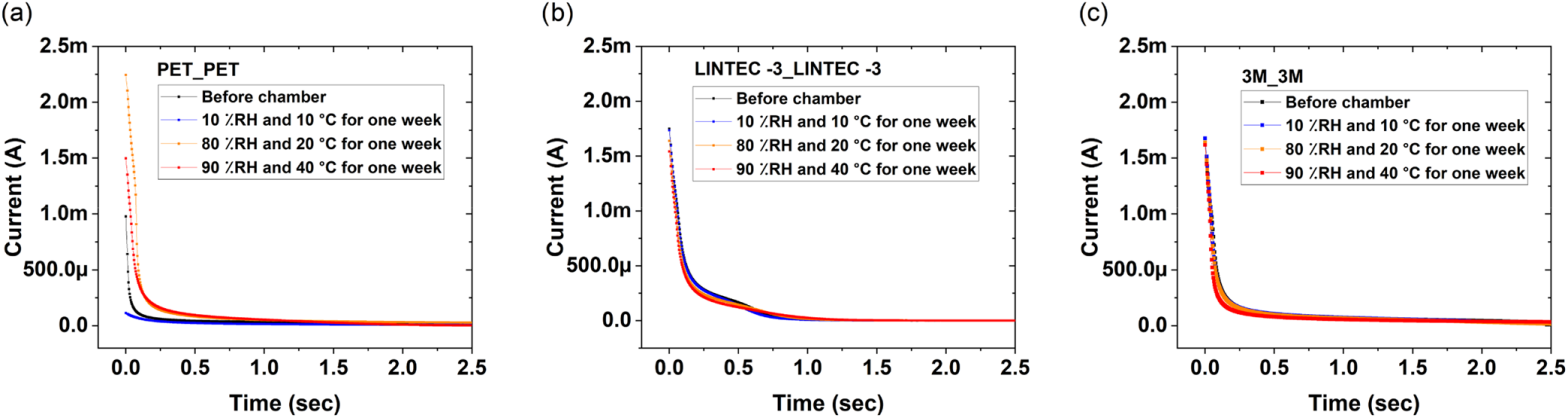
Current vs time switching behavior of the ring-shaped OECDs stored in different environmental conditions. The OECDs have PEDOT:PSS as both the top and bottom electrodes (Figure 3a), and the adhesive width is 15 mm. (a) OECDs encapsulated with PET are, as expected, clearly affected by the environmental changes; in principle, no coloration occurs upon storage in a cold and dry environment. (b) OECDs encapsulated with LINTEC-3 showed very similar switching behavior when stored in different environmental conditions. (c) OECDs encapsulated with 3M also showed a very similar switching behavior when comparing the current vs time curves after storing the devices in different environmental conditions.

OECDs printed on a PET substrate, followed by encapsulation with barrier films on both sides of the device sheet, also work very well, and it provides the possibility to encapsulate only when this is required by the specific application since the barrier films are expensive as compared to PET and other standard plastic substrates used for printed electronic applications.

Storage for an extended period of time at high temperature and high RH was also carried out for the OECD samples. The samples, which were printed on PET and encapsulated with LINTEC-3 and LINTEC-4, respectively, were stored at 90% RH and 40 °C for 56 days. Despite the harsh storage conditions, the OECDs still showed good switchability and color retention behavior. Further details on these tests are provided in Figure S9.

In addition to this, one OECD sample printed on PET and encapsulated with the LINTEC-3 barrier film was also tested for an extended period of 6 months at extremely dry conditions (<3% RH at 20 °C). The sample showed excellent switching behavior with no noticeable deterioration in terms of switching time and color contrast. More information is provided in Figures S10 and S11 as well as Movie S1.

### 3.3 Color Retention in Encapsulated OECD Devices

Color retention measurements of encapsulated OECD devices after 1 week of storage in the respective environmental conditions reveal that the LINTEC-4 barrier film combined with a 15-mm-wide adhesive frame gave the best result, while the LINTEC-3 barrier film with a 15-mm-wide adhesive frame was the second best option; see Figure 6. The difference could be due to several factors, but the barrier film properties may, of course, play a role since the LINTEC-4 barrier film exhibits better WVTR properties as compared to LINTEC-3. However, the color retention performance of OECDs encapsulated with 3M barrier films is worse as compared to devices encapsulated by both the LINTEC-3 and LINTEC-4 barrier films; the reason for this is unclear and could be due to different surface properties of the respective substrate. It should be pointed out that for the barrier films from LINTEC, the barrier coating is exposed to the printed layers, e.g., PEDOT:PSS, which minimizes diffusion of water vapor and oxygen, as well as that having the PET facing outward provides additional mechanical protection. For the barrier film from 3M, the printed layers are deposited onto PET since the barrier layer is embedded within the multilayered structure, which in turn may allow for diffusion of water vapor and oxygen through the cross-sectional area of the PET of the 3M barrier film upon long-time storage in harsh environmental conditions.

**Figure 6:**
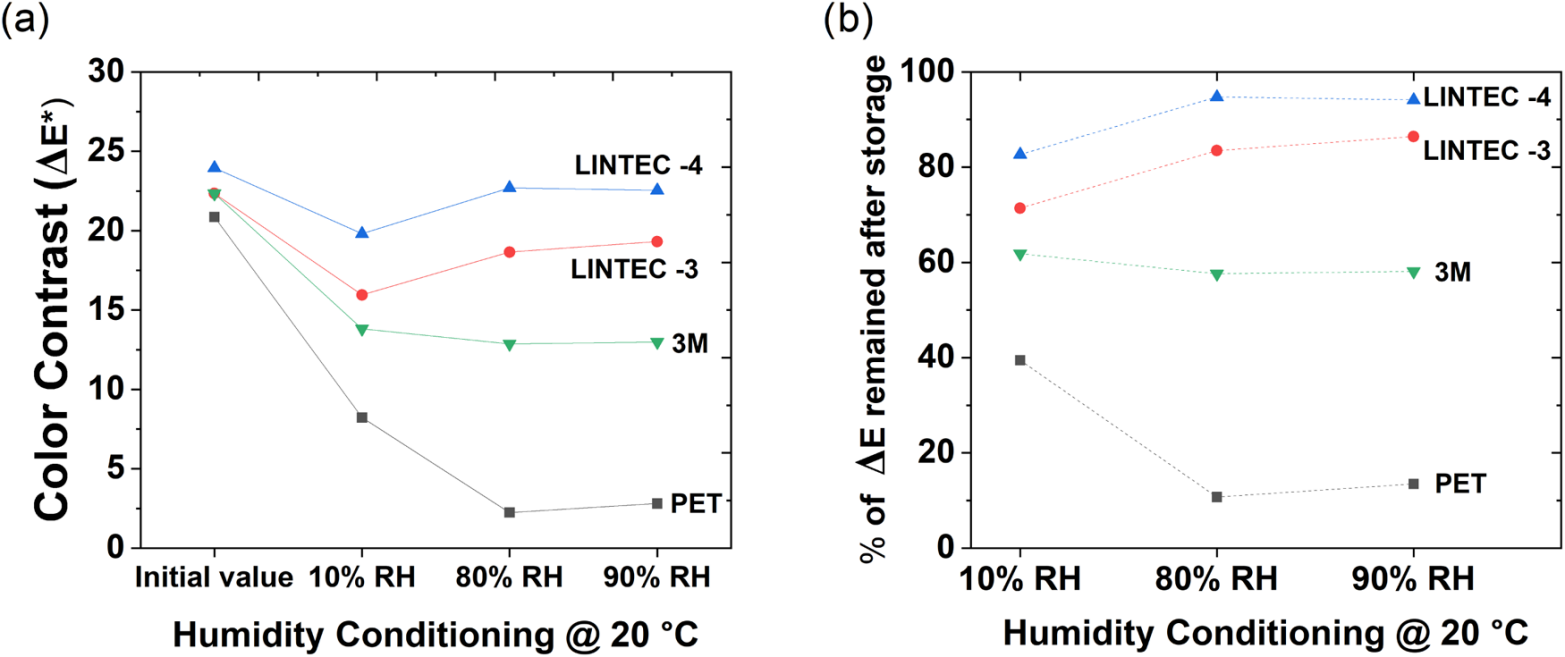
Color retention in OECDs encapsulated with different barrier materials after being stored in various environmental conditions. The OECDs have PEDOT:PSS as the bottom (electrochromic) electrode and carbon as the top (counter) electrode (Figure 3b). The display segments are first colored and then left in open-circuit mode for 1 week in each environmental condition before measuring the color contrast (ΔE*) again. (a) Devices encapsulated with PET show much lower color contrast values after storage; hence, their retention time is short. Devices encapsulated with LINTEC-4 barrier films and a 15-mm-wide adhesive layer show the best performance in terms of the retention time. Note that the color contrast of a nonencapsulated OECD would decay within hours, while the color contrasts of the best devices reported herein remain almost unaffected after 1 week of storage in various environmental conditions. (b) Data based on the measurements shown in panel (a), but here the remaining color contrast after storage is expressed in percentage.

## 4 Conclusions

Overall, proper device encapsulation can mitigate the influence of the environment on the devices, thereby ensuring good switching performances for both OECTs and OECDs. Here, screen-printed adhesive layers have successfully been used in the development of a scalable encapsulation process of these electrolyte-based screen-printed electronic components. The issue with air pockets, which occurs when laminating barrier films with precoated global adhesive layers, is avoided by instead depositing screen-printed adhesive layers prior to completing the encapsulation process by lamination.

Using the screen-printed adhesive layers results in conformal and leveled edge seals, which is a prerequisite for further evaluation of different types of barrier films. Storage tests have been carried out in a climate chamber, whereafter excellent results have been achieved for both OECDs and OECTs when using LINTEC-3, LINTEC-4, and 3M barrier films in the encapsulation process. Reference samples, in which PET was used as the barrier film, were used for comparison, and as expected, their switching performances were severely affected upon storage in different environmental conditions. In terms of providing a stable performance for the OECTs, 3M seems to be the best choice. This is also an expected result since the 3M barrier film exhibits the lowest WVTR value among the evaluated barrier films. The 3M barrier film is also mechanically robust, but its relatively high thickness (200 μm) results in less flexible devices. OECDs are less sensitive to different environmental conditions, as evidenced by the fact that barely any difference could be observed when comparing the barrier films in this evaluation; i.e., OECDs encapsulated with either LINTEC-3 or 3M barrier film performed equally well. The encapsulation process also provides considerable enhancement of the OECD color retention, and in this test, the LINTEC-4 barrier film provided the best performance. The LINTEC barrier films also exhibit high transmittance and the lowest haze values, and they also allow for highly flexible screen-printed electronic devices. However, their thinness (50 μm) makes the manufacturing more vulnerable to mechanical stress caused by the device topography and the lamination process, as shown by the difficulty in maintaining a high manufacturing yield while completing the encapsulation process by laminating a barrier film on top of an OECT screen-printed directly on the same kind of barrier film.

To conclude, the conformability of the screen-printed adhesive layers to both the substrate and the screen-printed silver lines efficiently prevents the problem of moisture ingress/egress that occurs to/from screen-printed OECTs and OECDs when using barrier films with a precoated global adhesive layer, and proper encapsulation by using screen-printed adhesive layers also enhances the color retention of the OECDs. In addition to this, readily available screen-printable adhesive materials and the development of a scalable process imply that the proposed encapsulation method may be used to improve other types of organic and printed electronic devices, such as electrochromic displays based on other material combinations, supercapacitors, and batteries, since they are all relying on electrolytes and similar device architectures,^66,67^ as well as OLEDs, OPVs, and OFETs.

## Supplementary Material





## Data Availability

The data underlying this study are openly available in Zenodo at https://doi.org/10.5281/zenodo.16583124.

## Notes

Movie S1 shows an almost unaffected OECD, despite it was stored in a dry chamber (<3% RH, 20 °C) for more than 6 months (MOV)
Photos of encapsulated OECT devices (Figure S1); OECT gate currents (Figure S2); simplified estimation of the relative impact on the water transmission rate for different adhesive widths and barrier films in encapsulated OECT devices (Figures S3, S4 and Tables S1, S2); OECTs with suppressed gate currents (Figure S5); humidity-dependent threshold voltage (*V*_TH_) in nonencapsulated OECTs (Figure S6); estimation of transmitted water vapor in encapsulated OECDs (Figures S7, S8 and Table S3); OECDs stored for 56 days in high RH and high temperature (Figure S9); encapsulated OECD stored for 6 months in very low RH and room temperature (Figures S10 and S11) (PDF)
