# Supplementary material for: Encapsulation of Screen-Printed Electrolyte-Based Organic Electronic Components for Long-Term Operation in Varying Environmental Conditions
Source: ACS Appl Mater Interfaces. 2025 Aug 5;17(32):45978–89. doi: 10.1021/acsami.5c09639 (PMC12356541; doi:10.1021/acsami.5c09639)
Supplement: Supplementary file 2 [file am5c09639_si_002.pdf]

Supporting Information (SI)

Encapsulation of Screen-Printed Electrolyte-Based Organic Electronic Components for Long-Term Operation in Varying Environmental Conditions

Xin Wang,[a]* Kathrin Freitag,[a] Jessica Åhlin,[a] and Peter Andersson Ersman[a]*

[a] Printed, Bio- and Organic Electronics – Smart Hardware – Digital Systems, RISE Research Institutes of Sweden, Södra Grytsgatan 4, SE-602 33 Norrköping, Sweden

*Corresponding authors: Dr. Xin Wang, Email: xin.wang@ri.se, ORCID: https://orcid.org/0000-0002-1182-4051; Dr. Peter Andersson Ersman, Email: peter.andersson.ersman@ri.se, ORCID: https://orcid.org/0000-0002-4575-0193

Photos of encapsulated OECT devices

Figure S1 shows photographs of the encapsulated OECT devices, where the sheets are laminated by using either 5 mm or 15 mm wide screen printed adhesive layers.

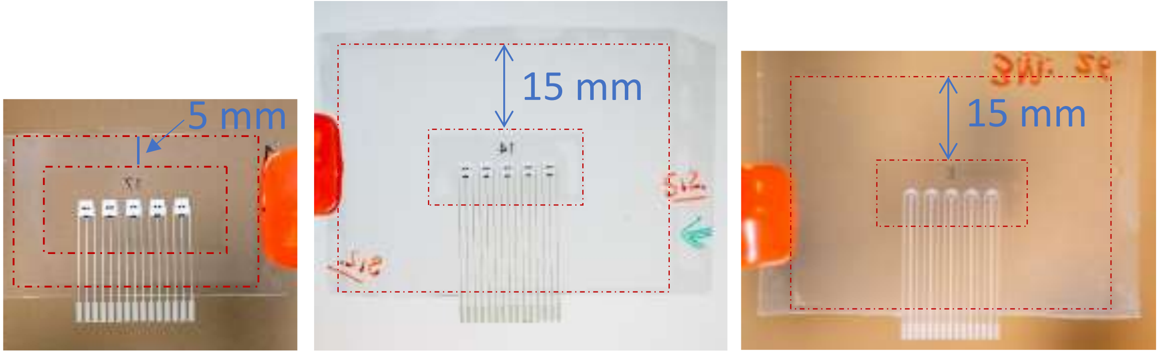

Figure S1. Photographs showing the encapsulated OECTs. No visible defects, e.g., bubbles, can be observed in the laminated structures, which is an indication that tight interfaces have been formed between the different surfaces that are joined together. The photographs also show the two different widths (5 mm and 15 mm) of the screen printed adhesive layer that have been used for the encapsulation process.

OECT gate currents

Figure S2 shows the gate currents for the different kinds of OECTs after storage at different environmental conditions; the data originate from the measurements shown in Figure 4. The increase of the gate current at elevated gate voltages is explained by the increased current between the gate and the drain electrodes. At these gate voltages the current between the source and drain electrodes is suppressed due to the reduced OECT channel, and the gate current level is dependent on the contribution from the water splitting reactions occurring at the carbon-based electrodes. Additionally, the graphs also show the dependency between the environmental condition and the respective barrier material; barrier films with low WVTR clearly mitigates the influence from various environmental conditions, where 3M seems to be the best option. The gate currents for the 3M-PET-3M samples are slightly higher as compared to the 3M-3M samples, even though both device types show very stable performance with respect to the different environmental conditions. The most plausible explanation for this is that these devices were not encapsulated at the same time, which may have resulted in slightly different environmental conditions during the preconditioning step.

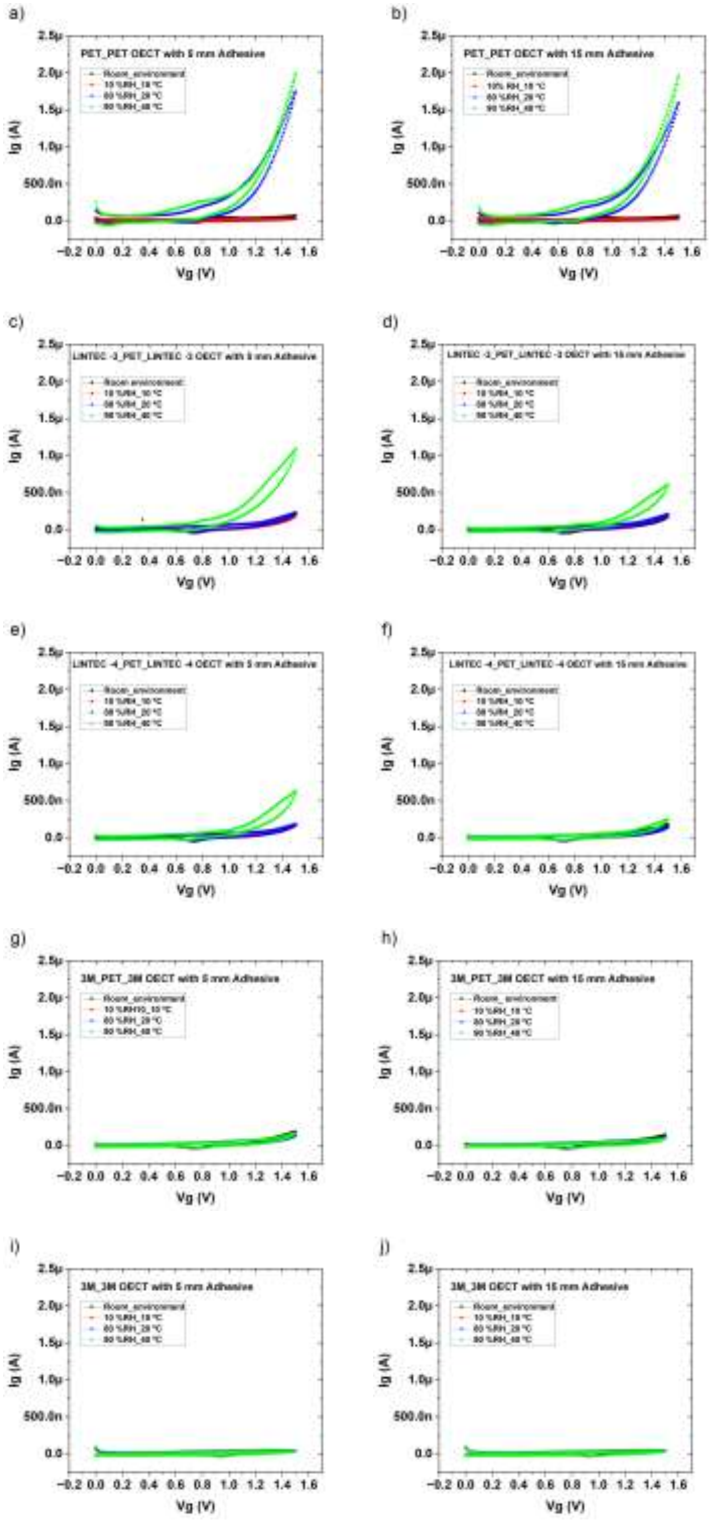

Figure S2. The gate currents of the OECT measurements shown in Figure 4. The gate current levels are affected by the barriers films that are used. a-b) The gate currents (Ig) for OECTs printed and encapsulated with PET "barrier film" and with either 5 or 15 mm wide adhesive layers, respectively; c-d) OECTs printed on PET and encapsulated with LINTEC -3 barrier film on both sides, and with either 5 or 15 mm wide adhesive layers, respectively; e-f) OECTs printed on PET and encapsulated with LINTEC -4 barrier film on both sides, and with either 5 or 15 mm wide adhesive layers, respectively; g-h) OECTs printed on PET and encapsulated with 3M barrier film on both sides, and with either 5 or 15 mm wide adhesive layers, respectively; i-j) OECTs printed on 3M and encapsulated with 3M barrier film on top of the printed layers, and with either 5 or 15 mm wide adhesive layers, respectively.

Simplified estimation of the relative impact on the water transmission rate, for different adhesive widths and barrier films in encapsulated OECT devices

The water vapor transmission for different parts/areas of the encapsulated device structures is defined as:

Area × WVTR

For the adhesive material, the outer cross section area is considered as the main water transmission route, the parts covered by the barrier films are omitted since water needs to overcome both the barrier film and the adhesive layer.

For the barrier film, the inner part, in which the adhesive is excluded, is defined as the main path for water ingress; the outer part that includes the adhesive layer in between is omitted in this estimation, for the same reason mentioned above. The dimensions of the encapsulated device structures are shown in Figure S3.

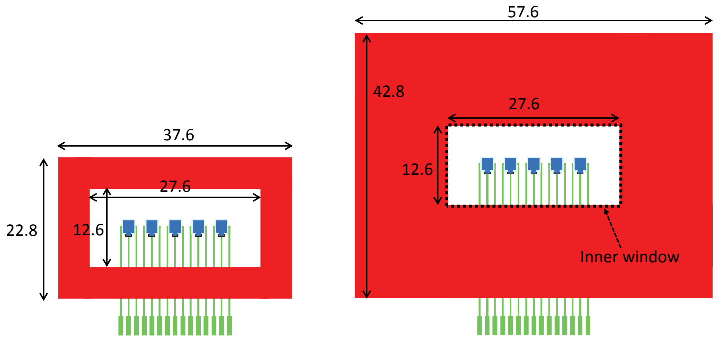

Figure S3. The layout and overall dimensions of the encapsulated screen printed OECTs used in this study. The unit for the dimensions of the schematics is mm. The adhesive material is marked in red color.

The WVTR parameter is thickness dependent, however, the WVTR of the adhesive material is unknown. A first assumption is to assign it the same value as the PET material, which has a relatively high WVTR. Since the water vapor transmission through the adhesive material occurs laterally along the width of the adhesive frame, the WVTR value needs to be adjusted to the corresponding thickness/width. Table S1 shows the thickness dependent WVTR for a material with either identical, 2.5 times higher or 4 times lower WVTR property as compared to PET.

Table S1. The table shows the thickness dependence of the WVTR material property.

	125 µm WVTR ($g \cdot m^{-2} \cdot day^{-1}$)	5 mm WVTR ($g \cdot m^{-2} \cdot day^{-1}$)	15 mm WVTR ($g \cdot m^{-2} \cdot day^{-1}$)
Material alike PET	4	0.1	0.033
Material with 4 times lower WVTR than PET	1	0.025	0.0083
Material with 2.5 times higher WVTR than PET	10	0.25	0.083

Water vapor transmission through the adhesive material is thus limited by the combined effects of the small cross-section area of the adhesive material and the relatively lower WVTR values determined by the long diffusion path due to the width of the screen printed adhesive material.

The amount of transmitted water vapor for a given device area (Area × WVTR) is calculated for the two routes: through the cross-section of the adhesive, assuming different WVTR values due to varying adhesive widths, and through the inner part of the barrier film areas not covered by the adhesive layer. The results are listed in Table S2, along with the dimensions of the device structures.

Table S2. OECT device dimensions and their estimated relative water permeation values.

	OECT (5 mm adhesive width)	OECT (15 mm adhesive width)
Outer length (mm)	37.6	57.6
Outer width (mm)	22.8	42.8
Thickness (adhesive) (mm)	0.026	0.026
Outer cross section area for adhesive (mm^2)	3.14	5.22
Area (of the outer cross section) × WVTR, assuming the adhesive has the same WVTR as PET with the same thickness of 5 or 15 mm ($\mu g\ day^{-1}$)	0.314	0.172
Area (of the outer cross section) × WVTR, assuming 0.25xWVTR as PET ($\mu g\ day^{-1}$)	0.0785	0.0431
Area (of the outer cross section) × WVTR, assuming 2.5xWVTR as PET ($\mu g\ day^{-1}$)	0.785	0.431
Inner length (mm)	27.6	27.6
Inner width (mm)	12.6	12.6
Inner "window" area (mm^2)	347.8	347.8
Area × WVTR LINTEC -3 (0.006) (inner_"window") ($\mu g\ day^{-1}$)	2.09	2.09
Area × WVTR LINTEC -4 (0.0005) ($\mu g\ day^{-1}$)	0.174	0.174
Area × WVTR 3M (0.00006) ($\mu g\ day^{-1}$)	0.0209	0.0209
Electrolyte area (for OECTs with large gate electrodes shown in Figure 4 and Figure S2) (mm^2)	9.0	9.0
Electrolyte area (for OECTs with small gate electrodes shown in Figure S5) (mm^2)	1.8	1.8

The results are plotted in Figure S4. From the calculations, it can be revealed that LINTEC -3 overshadows the influence of water vapor transmission through the adhesive material, which agrees well with the observations from the OECT measurements, where the performance of the OECTs with LINTEC -3 barrier films with either 5 or 15 mm wide adhesive frames are rather similar. For OECTs with LINTEC -4 and 3M barrier films, the 15 mm wide adhesive frames allow for lower amount of water vapor transmission, and this, together with the reduced amount of water vapor transmission through the LINTEC -4 and 3M barrier films, provide a good explanation to the improved results that were obtained. The results also indicate that the WVTR of the adhesive is at least similar to that of a PET material having the same dimensions, or lower, since this would provide a proper explanation to the switching performance of OECTs encapsulated with either LINTEC -4 or 3M barrier films.

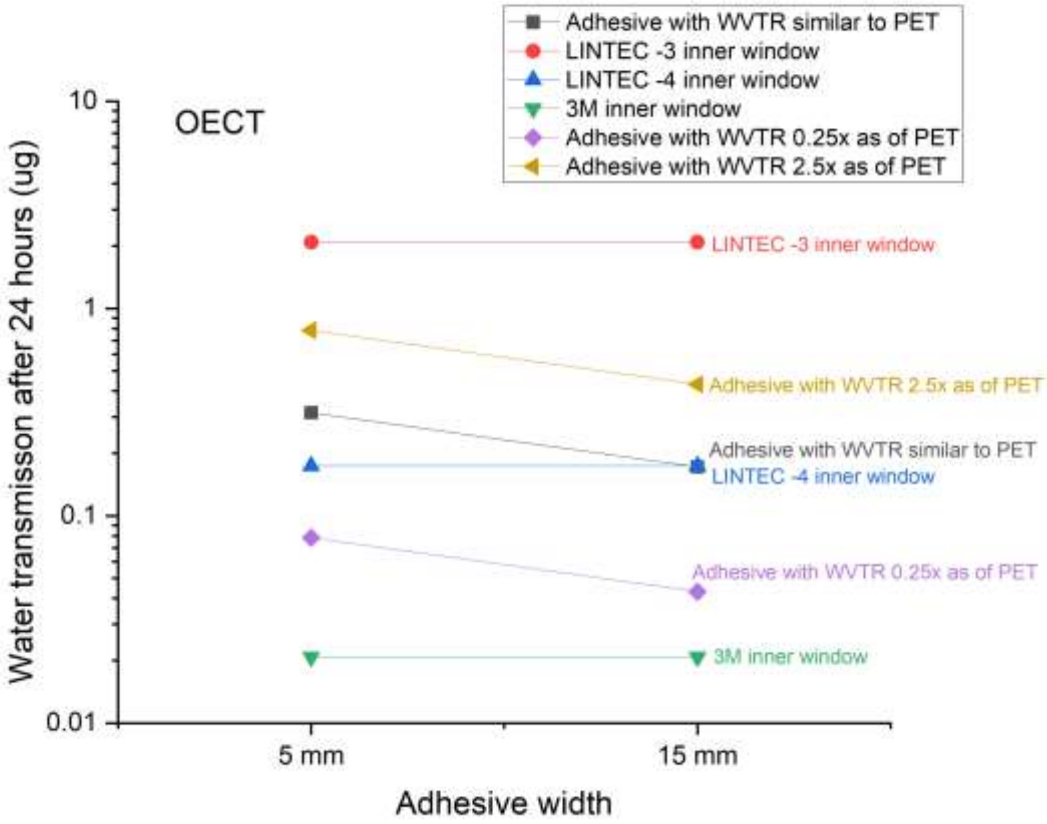

Figure S4. Water vapor transmission per day in encapsulated OECTs, for the respective barrier film, for different adhesive widths, for different assumptions of the WVTR values of the adhesive layers, and when only considering the inner window of the barrier film.

It should be noted that these calculations are simplified and only provide indications. More careful quantitative analyses and simulations would be a research topic in itself, which in turn would aid further optimization of the device designs. The WVTR of the adhesive material also needs to be measured in a controlled way, since such data is unavailable at present time. Additionally, it would be worthwhile to test adhesives with lower WVTR values, provided that they also are screen printable. It should also be noted that device architectures according to Figure 3d contain two barrier films, one on the front side and one on the back side, and correspondingly two adhesive layers, whereas devices built according to Figure 3e contain two barrier films but only one adhesive layer.

OECTs with suppressed gate currents

The parasitic gate current contribution is related to the areas of the carbon-based source and drain electrodes that are exposed to the electrolyte. The argument is further verified by another OECT architecture having smaller carbon areas in contact with the electrolyte, the measurement results of such OECTs are shown in Figure S5. As expected, the gate current levels in Figure S5 are lower than those shown in Figure S2.

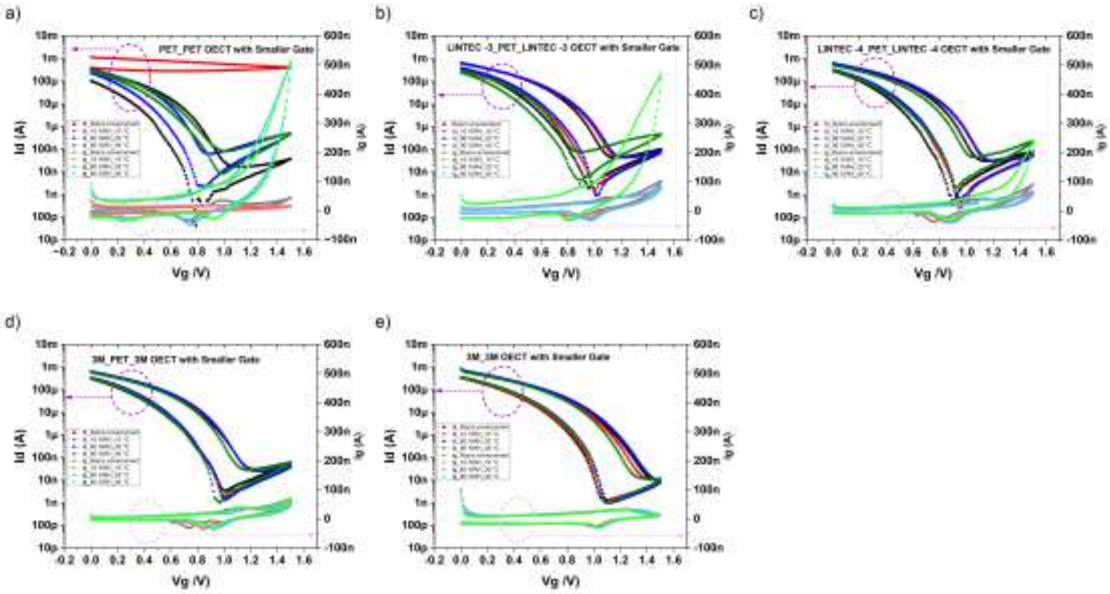

Figure S5. The current between the source and drain electrodes as well as the current between the gate and drain electrodes are shown. The carbon area exposed to the electrolyte is relatively smaller in these OECTs, as compared to the ones shown in Figure 4, thereby resulting in lower gate current levels. The edge sealing of the encapsulated OECTs were obtained by a 5 mm wide screen printed adhesive layer. The drain and gate currents (I_D and I_G) for a) OECTs printed and encapsulated with PET "barrier film"; b) OECTs printed on PET and encapsulated with LINTEC -3 barrier film on both sides; c) OECTs printed on PET and encapsulated with LINTEC -4 barrier film on both sides; d) OECTs printed on PET and encapsulated with 3M barrier film on both sides; e) OECTs printed on 3M and encapsulated with 3M barrier film on top of the printed layers.

Humidity dependent threshold voltage (V_{TH}) in non-encapsulated OECTs

By placing a non-encapsulated OECT sample in the climate chamber, the Vth value for the OECT was measured *in situ* as a function of temperature and humidity conditions. The results are shown in Figure S6.

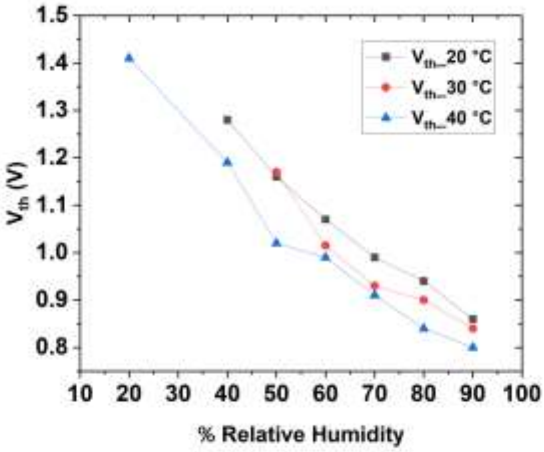

Figure S6. The V_{TH} in a non-encapsulated OECT is measured at different RH levels and temperatures. The V_{TH} is relatively independent of temperature fluctuations, but instead shows a very strong dependency upon varying the RH levels.

Estimation of transmitted water vapor in encapsulated OECDs

A similar approach for the estimation of water vapor transmission in encapsulated OECTs were carried out also for the encapsulated OECDs. Figure S7, Table S3 and Figure S8 show the device layouts, the dimensions of the adhesive layers and the estimated amount of transmitted water vapor in encapsulated OECDs. The dimensions of the OECDs and the accompanied encapsulation structures are slightly larger, however, simultaneously the electrolyte areas of the OECDs are also much larger, as compared to the OECTs, which provide them the ability to absorb/desorb more water without deteriorating. This could possibly explain why the switching performances of OECDs with LINTEC -3 and 3M barrier films were comparable, despite the large difference in WVTR between these two barrier films.

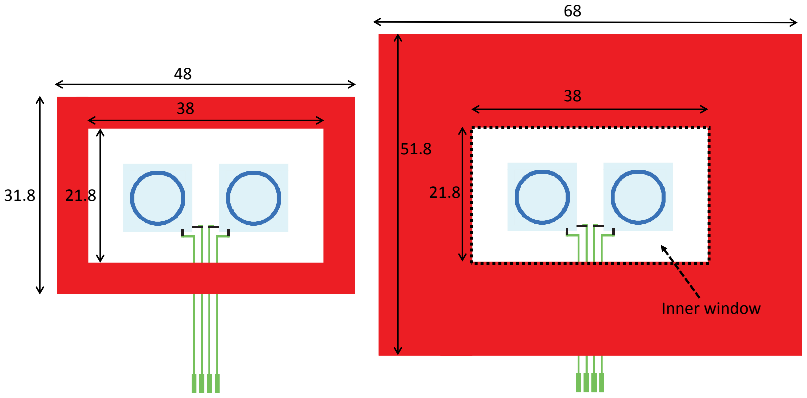

Figure S7. Top view of the OECD layouts, including the dimensions of the adhesive layers used in the encapsulation process. The unit of the dimensions is in mm. The adhesive material is marked in red color.

Table S3. Dimensions and water vapor transmission in encapsulated OECDs.

	OECD (5mm adhesive width)	OECD (15mm adhesive width)
Outer length (mm)	48	68
Outer width (mm)	31.8	51.8
Thickness (adhesive) (mm)	0.026	0.026
Outer cross section area for adhesive (mm^2)	4.15	6.23
Area (of the outer cross section) × WVTR, assuming adhesive having the same WVTR as PET with the same thickness of 5 or 15 mm (µg day^{-1})	0.415	0.206
Area (of the outer cross section) × WVTR, assuming 0.25xWVTR as PET (µg day^{-1})	0.104	0.0514
Area (of the outer cross section) × WVTR, assuming 2.5xWVTR as PET (µg day^{-1})	1.037	0.514
Inner length (mm)	38	38
Inner width (mm)	21.8	21.8
Inner “window” area	828.4	828.4
Area × WVTR LINTEC -3 (0.006) (inner_”window”) (µg day-1)	4.97	4.97
Area × WVTR LINTEC -4 (0.0005) (µg day-1)	0.414	0.414
Area × WVTR 3M (0.00006) (µg day -1)	0.0497	0.0497
Electrolyte area (mm^2)	200	200

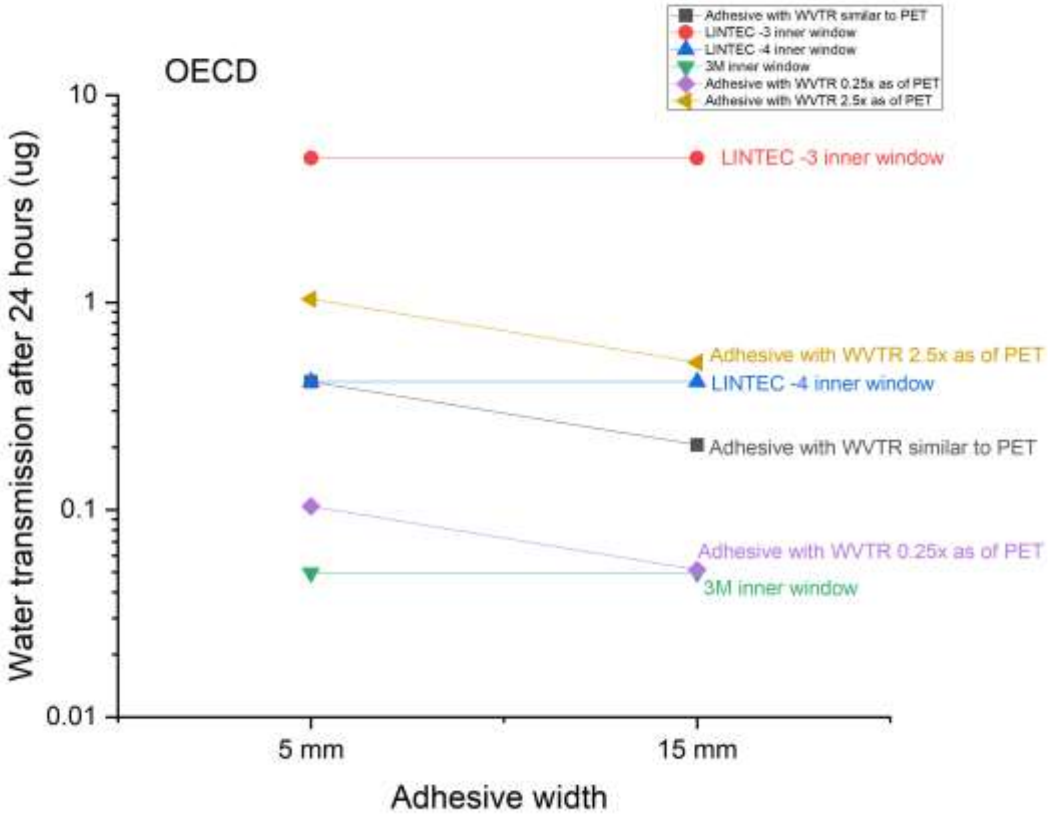

Figure S8. Water vapor transmission per day in encapsulated OECDs, for the respective barrier film, for different adhesive widths, for different assumptions of the WVTR values of the adhesive layers, and when only considering the inner window of the barrier film.

OECDs stored for 56 days in high RH and high temperature

OECDs screen printed on PET and then encapsulated with either LINTEC -3 or LINTEC -4 barrier films on both sides are shown in Figure S9. They work equally well as compared to the OECDs screen printed directly on barrier films. The samples were stored in a harsh condition for an extended period; 40 °C and 90 %RH for 56 days. The photographs in Figure S9 show the display samples after switching them to their reduced (ON) state and the color retention behavior 3 minutes after switching, revealing no discernable color change while kept in open-circuit mode during this time.

a)

Direct after switching 3 minutes after switching

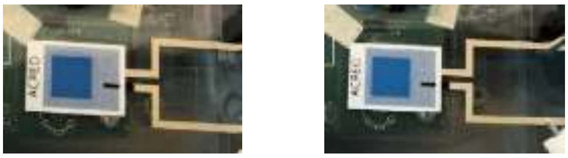

b)

Direct after switching 3 minutes after switching

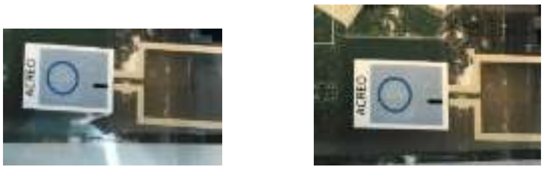

Figure S9. OECDs encapsulated with Lintec barrier films after long time (56 days) storage at high temperature (40 °C) and high relative humidity (90 %RH). a) An OECD encapsulated with LINTEC -3 barrier film, the respective photograph shows the appearance immediately after switching and the color retention behavior in open-circuit mode ≈3 minutes after switching. b) An OECD encapsulated with LINTEC -4 barrier film, the respective photograph shows the appearance immediately after switching and the color retention behavior in open-circuit mode ≈3 minutes after switching.

OECDs stored for 6 months in very low RH and room temperature

The sample was printed on PET and encapsulated with the LINTEC -3 barrier film. The electrical (current vs. time) measurements shown in Figure S10 were carried out *in situ* with the leads extending out from the dry chamber. The humidity inside the dry chamber dropped continuously until it stabilized at approximately 1.5 %RH. The dry chamber was operated at 20 °C.

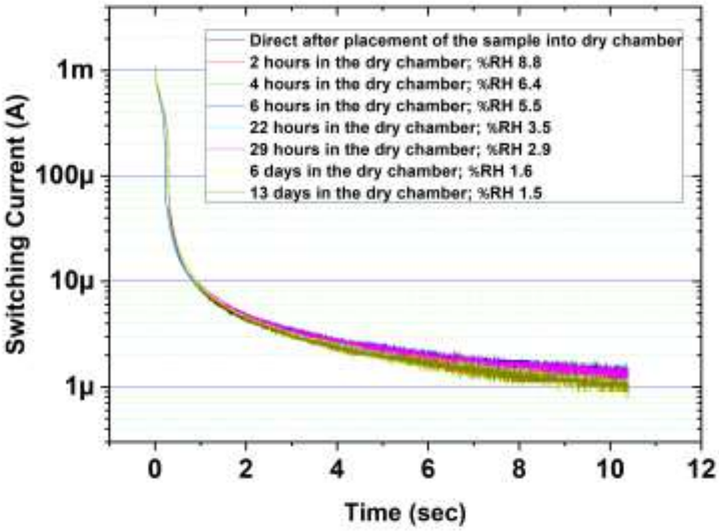

Figure S10. Current vs. time measurements of an OECD encapsulated with the LINTEC -3 barrier film. The OECD was stored for about 6 months at room temperature and an extremely dry condition (<3 %RH), even though the current vs. time data only were recorded for the first 13 days. The sample showed excellent switching behavior with no noticeable deterioration in terms of switching time and color contrast, see also Movie S1 and Figure S11.

Movie S1 shows an almost unaffected OECD, despite it was stored in a dry chamber (<3 %RH, 20 °C) for more than 6 months. The OECD was screen printed on a PET substrate and encapsulated with LINTEC -3 barrier films from both sides of the PET-based device substrate, see also Figure S11.

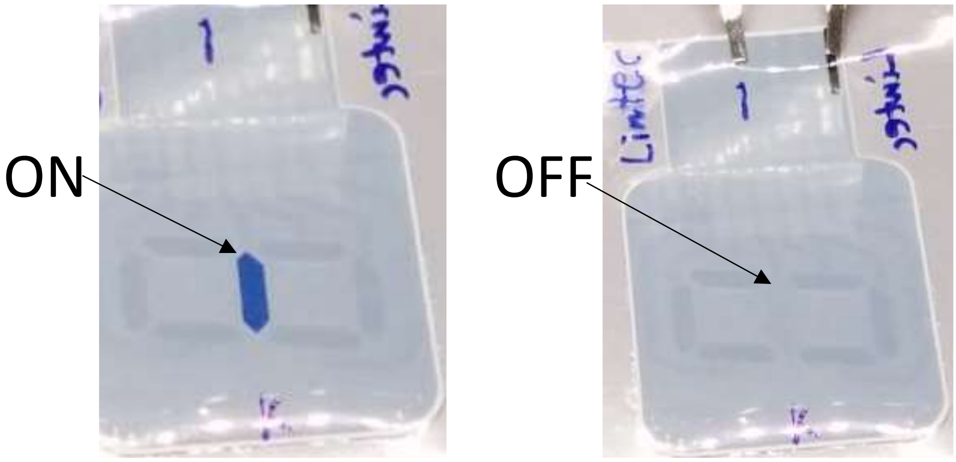

Figure S11. Photographs showing one OECD segment switched between its ON (left) and OFF (right) states after storage in <3 %RH and 20 °C for more than 6 months. The high color contrast indicates that the OECD is still fully operational, which is further evidenced by the switching response shown in Movie S1.
